# Risk factors associated with cesarean section and adverse fetal outcomes in intrahepatic cholestasis of pregnancy

**DOI:** 10.3389/fped.2023.1136244

**Published:** 2023-06-30

**Authors:** Chengcai Kong, Zonghao Zhu, Fenglin Mei

**Affiliations:** ^1^ Changzhou Maternal and Child Health Care Hospital, Changzhou Medical Center, Nanjing Medical University, Changzhou, China; ^2^ Department of Gynecology and Obstetrics, Third Affiliated Hospital of Suzhou University, Changzhou, China; ^3^ Department of Nursing, the Affiliated Changzhou NO.2 People's Hospital of Nanjing Medical University, Changzhou, China

**Keywords:** intrahepatic cholestasis of pregnancy, adverse fetal outcomes, cesarean section, preeclampsia, serum bile acids

## Abstract

**Background:**

To determine the risk factors for cesarean section (CS) and adverse fetal outcomes (AFOs) in patients with intrahepatic cholestasis of pregnancy (ICP) based on the severity of maternal hypercholanemia.

**Methods:**

A hospital-based retrospective cohort study was performed between January 1, 2015, and December 31, 2019. A total of 227 nulliparous women with a singleton fetus complicated by ICP were included. The patients were divided into two groups according to the levels of total bile acids, that is, mild (10 *μ*mol/L < total bile acids < 40 *μ*mol/L) and severe (≥40 *μ*mol/L). The patients' clinical characteristics and fetal outcomes were assessed.

**Results:**

Among the 227 eligible women, 177 (78.0%) were allocated to the mild group and 50 (22.0%) were in the severe group. Women with severe ICP also had a significantly higher incidence of planned and unplanned CS compared with mild ICP subjects (52.0% vs. 23.7% and 22.0% vs. 6.8%, respectively; *p* < 0.001). The indications for CS showed that fetal intolerance (65.4% vs. 14.3%) was higher in severe ICP compared with mild ICP (*p* < 0.001). Severe ICP was associated with an increased risk of preterm delivery (*p* < 0.001), low birthweight (*p* = 0.001), and neonatal intensive care unit (NICU) admission (*p* < 0.001). Women with severe ICP (OR 6.397, 95%CI 3.041–13.455, *p* < 0.001) or preeclampsia (OR 12.434, 95%CI 5.166–29.928, *p* < 0.001) had increased risks of AFOs compared to controls.

**Conclusions:**

Severe ICP and preeclampsia are associated with a higher incidence of AFOs.

## Introduction

Intrahepatic cholestasis of pregnancy (ICP) is characterized by unexplained maternal pruritus, raised serum bile acids (>10 μmol/L) and/or elevated liver transaminases (ALT > 35 U/L and/or AST > 35 U/L) during the late second and third trimester of pregnancy (1–3). Currently, the etiology of ICP is not fully understood but may be affected by hormonal, genetic, and environmental factors (4).

Although ICP was thought to be a relatively benign condition, recent studies have indicated that ICP has adverse effects for both the mother and fetus, including a notably higher risk of developing preeclampsia and gestational diabetes mellitus (GDM) (5, 6). Related to the fetus, ICP is associated with increased risks for preterm delivery (7), meconium staining of the amniotic fluid (MSAF) (8), neonatal intensive care unit admission (NICU) (3), and stillbirth (9), especially in cases of severe ICP.

According to previous studies, serum total bile acid levels >10 *μ*mol/L and <40 *μ*mol/L are considered mild ICP, while severe ICP is defined as maternal total bile acid levels ≥40 *μ*mol/L, which is associated with higher maternal and neonatal complications (3, 5). Glantz A, et al. recently demonstrated that adverse pregnancy outcomes did not occur until maternal serum bile acids were >40 *μ*mol/L (10). Previous studies reported that stillbirth in ICP tended to happen after 36 weeks of pregnancy (11). To prevent stillbirth, induction of labor has become common practice, especially in severe cases of patients. A large retrospective cohort study demonstrated that the risks of emergency caesarean section (CS) or fetal asphyxia were not increased after induction of labor in women with ICP during gestational weeks 37–39 (12), suggesting that this practice does not add additional risk. While the risks of adverse outcomes for both mothers and babies have started to be elucidated in women with ICP, the spectrum of ICP severity may differentially impact these risks and warrants further exploration.

The aim of this study was to evaluate maternal and fetal outcomes associated with severe ICP compared to mild ICP. Specifically, we set out to analyze potential differences in the indications for planned and unplanned CS and the rates of adverse fetal outcomes (AFOs) between women with severe ICP compared to women with mild ICP.

## Methods

The data for the retrospective cohort study were obtained from January 1, 2015, to December 31, 2019 at the Changzhou Maternal and Child Health Care Hospital in Jiangsu (China), where 10,000–12,000 deliveries per year are performed. Information on maternal demographics, medical comorbidities, and AFOs was obtained from medical records for evaluation. Women with fasting serum bile acid levels of more than 10 μmol/L and pruritus of unexplained cause were included in the study. Since multiparas and women with twin pregnancies might influence the mode of delivery directly, only nulliparous women with a singleton fetus were included in the study. Women were excluded if they had viral or autoimmune hepatitis, liver or biliary disease, and fetal chromosomal or structural abnormalities. The study protocol was approved by the Ethical Committee of the Hospital.

The patients were screened for ICP in pregnancy utilizing ICD- 10 code (O26.6). The women received ursodeoxycholic acid (UDCA) therapy, and fetal monitoring was performed more than once per day before delivery, except in cases where delivery occurred before ICP diagnosis. For the primary analysis, ICP severity was defined according to maternal total bile acids levels as mild (10 *μ*mol/L < total bile acids < 40 *μ*mol/L) or severe (≥40 *μ*mol/L).

Similar to previous studies (11, 13), the AFOs assessed included green staining of the placenta, an Apgar score of less than 7 at 5 min, preterm birth, placental abruption, neonatal unit admission, and stillbirth in this study. Preterm birth was defined as a gestational age before 37 completed gestational weeks. Information regarding the occurrence of GDM, gestational hypertension, preeclampsia, premature rupture of membranes (PROM), and other pathologies was also retrieved from the medical records.

Planned CS was defined as the intended mode of delivery, whereas all other CS were classified as unplanned. The indications for planned CS were severe preeclampsia, anatomic abnormality, estimated macrosomia, fetal intolerance of labor, and elective. The indications for unplanned CS were arrest of descent/dilation, non-reassuring fetal heart tracings (FHT), PROM + anatomic abnormality, and PROM + MSAF.

A two-tailed t-test was used to analyze continuous variables, and the *χ^2^* or Fisher's exact test was used to analyze categorical variables. A logistic regression analyse was also performed to assess the risks of AFOs. Our data met the calculated sample size for AFOs which was from 17 to 757 in each group for 80% and 90% power, and a 95% confidence interval. Statistical significance was defined as *p*-values < 0.05, and the data were analyzed using the statistical software package SPSS 22.0.

## Results

Two hundred twenty-seven pregnancies (177 mild and 50 severe ICP) met the inclusion criteria. The demographic and obstetric characteristics of the patients in the mild group and severe group are presented in Table 1. The analysis showed that maternal age, pregestational body mass index, and gestational diabetes mellitus, hypertension, preeclampsia, premature rupture of membranes, and meconium-stained amniotic fluid rate were not significantly different between the two groups (Table 1).

**Table 1:** Obstetric and demographic characteristics of patients with mild and severe intrahepatic cholestasis of pregnancy.

	Mild ICP	Severe ICP	*P* value
	(*n* = 177)	(*n* = 50)	
Maternal age (years)	26.7 ± 3.1	26.9 ± 3.4	0.684
pBMI (Kg/m^2^)	22.0 ± 3.6	21.1 ± 3.2	0.101
Gestational diabetes mellitus(%)	18 (10.2)	3 (6.0)	0.534
Hypertension(%)	8 (4.5)	2 (4.0)	1.000
Preeclampsia(%)	20 (11.3)	8 (16.0)	0.372
Premature rupture of membranes(%)	36 (20.3)	6 (12.0)	0.180
Meconium-stained amniotic fluid(%)	29 (16.4)	7 (14.0)	0.684
Iron-deficiency anemia(%)	32 (18.1)	6 (12.0)	0.309
Thrombocytopenia(%)	6 (3.4)	5 (10.0)	0.121
Oligohydramnios(%)	1 (0.6)	2 (4.0)	0.123ª
Small for gestational age(%)	2 (1.1)	2 (4.0)	0.211ª
Postpartum hemorrhage(%)	10 (5.6)	2 (4.0)	0.918
Cord around neck(%)	36 (20.3)	7(14.0)	0.312

A *t*-test was used to compare means, and the *χ^2^* or Fisher's exact test was used to analyze number (percentage).

^a^ Fisher's exact.

pBMI, pregestational body mass index.

There were significant differences in the mode of delivery found between women with severe ICP compared to those with mild ICP (*p* < 0.001, Table 2) where a greater proportion of women with severe ICP underwent CS. Furthermore, women with severe ICP had a higher frequency of unplanned CS compared to those with mild ICP (22% vs. 6.8%, *p* < 0.001). The indications for planned/unplanned CS in women with mild or severe ICP are shown in Table 3. Most planned CS for patients with severe ICP were due to fetal intolerance (65.4%) which had lower incidence (14.3%) in women with mild ICP (*p* < 0.001). However, the rate of the indications for unplanned CS was not significantly different between the two groups (*p* > 0.05).

**Table 2:** Mode of delivery in cases of mild and severe intrahepatic cholestasis of pregnancy.

	Mild ICP	Severe ICP	*P* value
	(*n* = 177)	(*n* = 50)	
Vaginal delivery	123 (69.5)	13 (26)	<0.001
Caesarean section			
Planned caesarean section	42 (23.7)	26 (52)	
Unplanned caesarean section	12 (6.8)	11 (22)	

The *χ^2^* test was used to analyze number (percentage).

**Table 3:** Indications for planned or unplanned caesarean section.

	Mild ICP	Severe ICP	*P* value
Planned caesarean section	*n* = 42	*n* = 26	
Severe preeclampsia	10 (23.8)	2 (7.7)	0.172
Anatomic abnormality	9 (21.4)	1 (3.8)	0.102
Estimated macrosomia	2 (4.8)	1 (3.8)	1.000
Fetal intolerance of labor	6 (14.3)	17 (65.4)	<0.001
Elective	15 (35.7)	5 (19.2)	0.147
Unplanned caesarean section	*n* = 12	*n* = 11	
Arrest of descent/dilation	5 (41.7)	3 (27.3)	0.667^a^
Non-reassuring FHT	3 (25)	4 (36.4)	0.667^a^
PROM + Anatomic abnormality	1 (8.3)	1 (9.0)	1.000^a^
PROM + MSAF	3 (25)	3(27.3)	1.000^a^

The *χ^2^* or Fisher's exact test was used to analyze number (percentage).

^a^ Fisher's exact.

FHT, fetal heart tracings; PROM, premature rupture of membranes; MSAF, meconium staining of the amniotic fluid.

We further analyzed AFOs between the two groups (Table 4). A greater proportion and/or frequency of women with severe ICP had preterm delivery (40.0% vs. 8.5%, *p* < 0.001) and low birth weight (28.0% vs. 6.8%, *p* = 0.001) compared to those with mild ICP, Table 4. The incidence of NICU admission (8.5% vs. 32.0%, *p* < 0.001) and total AFOs (10.2% vs. 42.0%, *p* < 0.001) between mild ICP and severe ICP groups was significantly lower (Table 4).

**Table 4:** Birth weight and adverse fetal outcomes in mild and severe intrahepatic cholestasis of pregnancy.

	Mild ICP	Severe ICP	*P* value
	(*n* = 177)	(*n* = 50)	
Green staining of the placenta(%)	2 (1.1)	3 (6.0)	0.127
Placental abruption(%)	1 (0.6)	1 (2.0)	0.393ª
Delivery gestational age(%)			
≥37 weeks	162 (91.5)	30 (60.0)	<0.001
34 to 37 weeks	10 (5.7)	16 (32.0)	
≤34 weeks	5 (2.8)	4 (8.0)	
Birth weight(%)			
<2,500 g	12 (6.8)	14 (28.0)	0.001
2,500 to 4,000 g	155 (87.6)	34 (68.0)	
>4,000 g	10 (5.7)	2 (4.0)	
Fetal asphyxia(%)	5 (2.8)	5 (10.0)	0.073
Stillbirth(%)	2 (1.1)	1 (2.0)	0.528ª
NICU admission(%)	15 (8.5)	16 (32.0)	<0.001
Total AFOs(%)	18 (10.2)	21(42.0)	<0.001

The *χ^2^* or Fisher's exact test was used to analyze number (percentage).

^a^ Fisher's exact.

AFOs, adverse fetal outcomes.

We discovered a strong association between severe ICP and AFOs (OR 6.397, 95%CI 3.041–13.455, *p* < 0.001) using a logistic regression model (Table 5). Among GDM, hypertension, preeclampsia, MSAF, and PROM, only women with preeclampsia were significantly more likely to have AFOs (OR 12.434, 95%CI 5.166–29.928, *p* < 0.001, Table 5).

**Table 5:** Ors for predictors of AFOs in intrahepatic cholestasis of pregnancy.

Variables	OR (95% CI)	*P* value
Severe ICP^a^	6.397 (3.041–13.455)	<0.001
Gestational diabetes mellitus	1.581 (0.543–4.606)	0.401
Hypertension	1.216 (0.248–5.960)	0.809
Preeclampsia	12.434 (5.166–29.928)	<0.001
PROM	0.600 (0.220–1.640)	0.320
MSAF	0.958 (0.369–2.484)	0.929

^a^ Reference category is mild ICP.

AFOs, adverse fetal outcomes; PROM, premature rupture of membranes; MSAF, meconium staining of the amniotic fluid.

## Discussion

The present data indicate that women with severe ICP have a greater proportion of CS, including planned and unplanned CS, and a higher incidence of AFOs, compared to women with mild ICP. Percentage of 51.1 more women with severe ICP had fetal intolerance of labor compared to women with mild ICP in planned CS. Preeclampisa might lead to an increase risk of AFOs both in mild and severe ICP.

Tetsuya Kawakita et al. reported that the rate of CS in women with mild or severe ICP was less than 40% (9). In our study, almost 30% of patients with mild ICP underwent CS, while 74% of patients with severe ICP underwent CS. Given that patient preference to choose CS to avoid stillbirth while with severe ICP, the increased incidence of CS in women with a total bile acids level ≥40 μmol/L is not unexpected. Moreover, another study revealed that women with ICP during gestational weeks 37–39 had no increased risk of emergency CS compared to women without ICP; however, this study was limited to gestational weeks 37–39 and lacked data regarding preterm birth (12). As many authors have advocated the implementation of elective early delivery for ICP, especially for severe ICP (11, 14), the rates of planned and unplanned CS in our patients with severe ICP were higher than patients with mild ICP.

Although there are no randomized studies investigating the optimal timing of delivery for women with ICP, a majority of researchers have advocated the implementation of elective early delivery for ICP, especially for those patients with total bile acids levels ≥40 *μ*mol/L (11). Most spontaneous or induced onset of early term labor did not increase the incidence of CS; however, many women with ICP in need of induction of labor underwent CS directly, which may have increased the incidence of CS in our study. Furthermore, to find the potential risk factors associated with CS, we analyzed the indication for these CS cases, which might diverge from current guidelines, containing planned and unplanned CS with different ICP severity. More than half of planned CS with severe ICP occurred due to fetal intolerance of labor, while most unplanned CS with severe ICP occurred for non-reassuring FHT, arrest of descent/dilation, and PROM + MSAF. Although the mechanism for the high rate of these events in severe ICP undergoing CS remains unclear, these indications were the reason the obstetrician chose planned or unplanned CS to prevent poor prognosis. Further research is needed to identify the ideal time of birth with regard to neonatal outcomes and to reduce the rates of CS if possible.

Recent studies have reported that an elevated total bile acids level may cause preeclampsia or GDM (5, 6). Preeclampsia occurs most often 2–4 weeks after the diagnosis of ICP, and still occurred even though total bile acids levels decreased toward the normal range according to a recent study from Raz et al. (5). Missing preeclampsia patients with a high level of total bile acids which returns to normal range later might leading to our study did not find a higher incidence of preeclampsia in women with severe ICP compared to mild ICP. Unexpectedly, our study did not find that GDM was concomitant with ICP severity at initial diagnosis, which is similar to that reported by Dan Shan, et al. (15). Advanced age and increased pregestational BMI may contribute more to the pathogenesis of GDM, in addition to the known effects on bile acid homeostasis.

Previous studies have reported that high bile acid levels could cause MSAF (8, 15). Moreover, previous reports have indicated that MSAF is more likely to occur during later compared to earlier gestation, which may be due to the higher bile acid concentrations in amniotic fluid and a more advanced maturation of the gastrointestinal tract (8). Women with severe ICP might reduce the incidence of MSAF by choosing to deliver before week 37. Although our study did not find MSAF to be associated with an increased risk of AFOs, obstetricians usually make decisions by considering the MSAF and the time to delivery to prevent newborns from developing meconium aspiration syndrome, which can be fatal.

As reported in previous studies, severe ICP as well as preeclampsia are risk factors of AFOs (16, 17), including preterm delivery, low birth weight, NICU admission, and stillbirth. After ORs for predictors of adverse fetal outcomes in ICP, we further found that preeclampsia was the risk factor of AFOs in both mild and severe ICP. Previous studies demonstrated that increased bile acids indeed crossed the placenta with increased oxidative stress contributing to the incidence of AFOs (18). Even if the relationship between preeclampsia and ICP is not considered, preeclampsia can also lead to AFOs through affecting the placental blood supply (19).

The present retrospective study has several limitations. Although the patients in our study were treated with UDCA, except for those who delivered before ICP diagnosis, the lack of data regarding the specific time and dose of UDCA use limited our ability to analyze the effect of UDCA on total bile acids levels. A previous study indicated that UDCA treatment appeared to improve the prognosis of both women with ICP and the fetus (20). However, Tetsuya Kawakita, et al. concluded that UDCA was not associated with a reduction in the risk of AFOs (9). Additionally, the number of patients with AFOs was small, which could have been a confounding factor. Since the study presented here was retrospective, we could not evaluate any rare outcomes such as intracranial hemorrhage, and sepsis.

## Conclusion

The present retrospective study demonstrates a significantly increased incidence of CS, including planned and unplanned, in women with severe ICP. Severe ICP and preeclampsia were associated with AFOs, suggesting that further prospective studies are warranted to identify the proper treatment to improve fetal outcome.

## Data Availability

The raw data supporting the conclusions of this article will be made available by the authors, without undue reservation.

## References

[1] Song X Vasilenko A Chen Y Valanejda L Verma R Yan B Transcriptional dynamics of bile salt export pump during pregnancy: mechanisms and implications in intrahepatic cholestasis of pregnancy. Hepatology. (2014) 60:1993–2007. 10.1002/hep.27171 24729004 PMC4194188

[2] Wang F He Y Yao N Ruan L Tian Z. High levels of serum superoxide dismutase as a biomarker of intrahepatic cholestasis of pregnancy in patients with viral hepatitis B. BMC Pregnancy Childbirth. (2022) 22:444. 10.1186/s12884-022-04776-y 35643465 PMC9145169

[3] Jin J Pan SL Huang LP Yu YH Zhong M Zhang GW. Risk factors for adverse fetal outcomes among women with early- versus late-onset intrahepatic cholestasis of pregnancy. Int J Gynaecol Obstet. (2015) 128:236–40. 10.1016/j.ijgo.2014.09.013 25468047

[4] Jurate K Rimantas Z Jolanta S Vladas G Limas K. Sensitivity and specificity of biochemical tests for diagnosis of intrahepatic cholestasis of pregnancy. Ann Hepatol. (2017) 16:569–73. 10.5604/01.3001.0010.0294 28611260

[5] Raz Y Lavie A Vered Y Goldiner I Skornick-Rapaport A Landsberg Asher Y Severe intrahepatic cholestasis of pregnancy is a risk factor for preeclampsia in singleton and twin pregnancies. Am J Obstet Gynecol. (2015) 213:395.e1–8. 10.1016/j.ajog.2015.05.011 25979617

[6] Liu C Gao J Liu J Wang X He J Sun J Intrahepatic cholestasis of pregnancy is associated with an increased risk of gestational diabetes and preeclampsia. Ann Transl Med. (2020) 8:1574. 10.21037/atm-20-4879 33437773 PMC7791254

[7] Pata O Vardarelı E Ozcan A Serteser M Unsal I Saruç M Intrahepatic cholestasis of pregnancy: correlation of preterm delivery with bile acids. Turk J Gastroenterol. (2011) 22:602–5. 10.4318/tjg.2011.0427 22287405

[8] Estiú MC Frailuna MA Otero C Dericco M Williamson C Marin J Relationship between early onset severe intrahepatic cholestasis of pregnancy and higher risk of meconium-stained fluid. PLoS One. (2017) 12:e0176504. 10.1371/journal.pone.0176504 28437442 PMC5402936

[9] Kawakita T Parikh LI Ramsey PS Huang CC Zeymo A Fernandez M Predictors of adverse neonatal outcomes in intrahepatic cholestasis of pregnancy. Am J Obstet Gynecol. (2015) 213:570.e1–8. 10.1016/j.ajog.2015.06.021 26071912 PMC5199141

[10] Glantz A Marschall HU Mattsson LA. Intrahepatic cholestasis of pregnancy: relationships between bile acid levels and fetal complication rates. Hepatology. (2004) 40:467–74. 10.1002/hep.20336 15368452

[11] Puljic A Kim E Page J Esakoff T Shaffer B LaCoursiere DY The risk of infant and fetal death by each additional week of expectant management in intrahepatic cholestasis of pregnancy by gestational age. Am J Obstet Gynecol. (2015) 212:667.e1–5. 10.1016/j.ajog.2015.02.012 25687562

[12] Wikström Shemer EA Thorsell M Marschall HU Kaijser M. Risks of emergency cesarean section and fetal asphyxia after induction of labor in intrahepatic cholestasis of pregnancy: a hospital-based retrospective cohort study. Sex Reprod Healthc. (2013) 4:17–22. 10.1016/j.srhc.2012.11.005 23427928

[13] Rook M Vargas J Caughey A Bacchetti P Rosenthal P Bull L. Fetal outcomes in pregnancies complicated by intrahepatic cholestasis of pregnancy in a northern California cohort. PLoS One. (2012) 7:e28343. 10.1371/journal.pone.0028343 22403605 PMC3293870

[14] Lo JO Shaffer BL Allen AJ Little SE Cheng YW Caughey AB. Intrahepatic cholestasis of pregnancy and timing of delivery. J Matern Fetal Neonatal Med. (2015) 28:2254–8. 10.3109/14767058.2014.984605 25371372

[15] Shan D Hu Y Qiu P Mathew BS Chen Y Li S Intrahepatic cholestasis of pregnancy in women with twin pregnancy. Twin Res Hum Genet. (2016) 19:697–707. 10.1017/thg.2016.74 27677539

[16] Usta A Turan G Sancakli Usta C Avci E Adali E. Placental fractalkine immunoreactivity in preeclampsia and its correlation with histopathological changes in the placenta and adverse pregnancy outcomes. J Matern Fetal Neonatal Med. (2020) 33:806–15. 10.1080/14767058.2018.1505854 30049235

[17] Greiner KS Speranza RJ Rincón M Beeraka SS Burwick RM. Association between insurance type and pregnancy outcomes in women diagnosed with hypertensive disorders of pregnancy. J Matern Fetal Neonatal Med. (2020) 33:1427–33. 10.1080/14767058.2018.1519544 30182768

[18] Wang J Lun W Shi W. Effects of elevated bile acid levels on fetal myocardium in intrahepatic cholestasis of pregnancy, a retrospective study from a neonatal perspective. Clin Res Hepatol Gastroenterol. (2022) 46:102013. 10.1016/j.clinre.2022.102013 36044978

[19] Licini C Avellini C Picchiassi E Mensà E Fantone S Ramini D Pre-eclampsia predictive ability of maternal miR-125b: a clinical and experimental study. Transl Res. (2021) 228:13–27. 10.1016/j.trsl.2020.07.011 32726711

[20] Joutsiniemi T Timonen S Linden M Suvitie P Ekblad U. Intrahepatic cholestasis of pregnancy: observational study of the treatment with low-dose ursodeoxycholic acid. BMC Gastroenterol. (2015) 15:92. 10.1186/s12876-015-0324-0 26215400 PMC4517361

